# Longitudinal evidence on the development of socioeconomic inequalities in mental health due to the COVID-19 pandemic in Norway

**DOI:** 10.1038/s41598-022-06616-7

**Published:** 2022-03-09

**Authors:** Bjørn-Atle Reme, Jonathan Wörn, Vegard Skirbekk

**Affiliations:** Center for Fertility and Health, Norwegian Institute of Public Health, Postbox 222 Skøyen, N-0213 Oslo, Norway

**Keywords:** Diseases, Signs and symptoms

## Abstract

The economic and health consequences of the COVID-19 pandemic are unequally distributed. A growing literature finds evidence that those with low socioeconomic status have carried a greater burden in terms of both unemployment and infection risk. Against this background, it is natural to also expect increasing socioeconomic inequalities in mental health. We report from a population-based longitudinal study, assessing the mental health of more than 100,000 Norwegian adults during a period of more than 20 years, and into the COVID-19 pandemic. We find substantial, and equally high, increases in depressive symptoms across socioeconomic status. In addition, we show that the increase was particularly strong among women and those with lower levels of depressive symptoms prior to COVID-19.

## Introduction

The COVID-19 pandemic has affected individuals’ physiological, psychological and material wellbeing around the world^1–4^. However, several studies suggest that these effects are not equally distributed. For example, a growing body of research finds that those with lower socioeconomic status (SES) were both more likely to be diagnosed with COVID-19 and more likely to lose their job compared to those with higher SES^5–8^. This lines up with research reporting that persons with lower SES experience higher health risks and higher economic risks during the pandemic^9^. Provided the well-documented impact of economic hardship on mental health, it is natural to also expect an increase in mental health inequalities across SES during the COVID-19 pandemic^10,11^.

A large literature documents substantial mental health differences across SES^12^. Particular attention has been given to depression, with empirical evidence pointing towards a negative association between SES and the likelihood of depression^13^. Hence, an important question for policy makers is how the COVID-19 pandemic has affected this association. The largest studies using longitudinal data, thus far, are based on the UK Household Longitudinal Study and cover 17,400 and 8200 individuals, respectively^2,14^. While these studies find a widening of mental health inequalities during the COVID-19 pandemic along dimensions such as age, gender and living with young children, they did not find changes in inequality along SES. At the same time, and contrary to speculations about the COVID-19 pandemic as a cause for widening inequalities, smaller studies find evidence suggesting a *decrease* in mental health inequalities. For example, a study based on a nationally representative sample of approximately 1100 US-Americans reports a larger increase in depressive symptoms among those with higher education between 2019 and the first wave of the pandemic in April 2020^15^. Moreover, a study on life satisfaction among Norwegian lower secondary school students reported less socioeconomic inequality during the period of closed schools in April and May 2020, compared to other assessments in 2020 taken prior to the introduction of measures to mitigate the spread of Sars-COV2^16^.

Against this background of inconclusive evidence and limited sample sizes in previous studies, we use longitudinal population level data from more than 100,000 mothers and fathers from Norway to provide evidence on how the COVID-19 pandemic affected socioeconomic inequalities in mental health. This study compares socioeconomic inequalities in depressive symptoms before the COVID-19 pandemic and during the first months of the COVID-19 pandemic in 2020. In addition, we examine how effects differ across the respondents’ mental health status prior to the COVID-19 pandemic. The evidence thus far on such effect heterogeneities is limited and based on cross-sectional or comparably smaller longitudinal samples^17–19^. Given that women on average have a higher risk of experiencing depression or reporting depressive symptoms than men^20^, we further take into account that the pandemic might affect mental health inequalities differently for men and women.

## Data and methods

### Norwegian mother, father and child cohort study and the NorFlu coronavirus study

The study is based on data from the The Norwegian Mother, Father and Child Cohort Study (MoBa), a population-based pregnancy cohort study conducted by the Norwegian Institute of Public Health^21^. The adult participants (approx. 170,400; 95,200 mothers and 75,200 fathers) were recruited from all over Norway in the period 1999–2008. The first MoBa questionnaire for female participants was administered in the 15th week of the pregnancy, with 10 follow-up surveys until the child is 14 years old. Fathers were surveyed in the 15th week of the pregnancy and a second time in 2015–2018. At the onset of the COVID-19 pandemic in Norway, 149,000 MoBa participants were invited to participate in an extended version of The Norwegian Influenza Study (NorFlu Coronavirus), which aimed at assessing the health consequences of COVID-19. Data collection for NorFlu Coronavirus began in week 14 of 2020 (starting March 30), 15 days after schools and day care centers were closed. Participants were invited for follow-up interviews every 14 days thereafter, and data collection was still ongoing as of May 2021.

**Figure 1:**
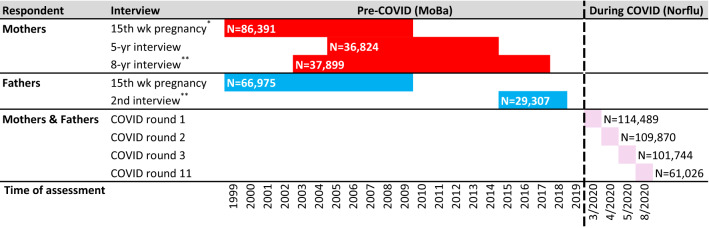
**Overview of survey waves and time-periods for data used in the analysis.** The table provides an overview of the different waves of the MoBa and NorFlu Coronavirus studies used in the analysis and the number of participants in the raw data. All these waves contained the measures of depressive symptoms SCL-5. Interview waves marked with *contain data on both education and income. Interview waves marked with **contained data on education. Dates for NorFlu Coronavirus rounds are given in approximate calendar dates, with exact dates being March 31–April 14 (NorFlu Coronavirus round 1), April 14–April 29 (round 2), April 29–May 12 (round 3) and August 19–September 1 (round 11).

Figure 1 below illustrates when information on variables used in this study were collected. Education could be reported at multiple occasions, and the highest reported level of education was used in the analyses. Income was reported once, specifically in the 15th week of pregnancy for mothers and in 2015–2017 for fathers. Mental health, as measured by the Hopkins Symptom Checklist-5 (SCL-5), was obtained in all the MoBa-interviews used in this study and in four interviews of NorFlu between April and August 2020, specifically during the following periods: March 31–April 14 (NorFlu Coronavirus round 1), April 14–April 29 (round 2), April 29–May 12 (round 3) and August 19–September 1 (round 11). SCL-5 was not assessed in rounds 4 to 10. Table A.1 in the Supplementary Material provides descriptive statistics of the sample and a more detailed overview of the data used in the study.

### Sample

Our main analytical sample was comprised of 202,024 observations from 111,294 respondents. Among these, 88,041 respondents participated in at least one of the utilized NorFlu Coronavirus waves. Across all person-observations, average age in 2020 was 46.4 years (SD = 5.3). Furthermore, 26% of observations were from male participants and 68% of observations from persons who had university education (see Tables A.1 and A.2 in the supplementary materials for more descriptive statistics).

### Analytical strategy

We estimated mean values and 95%-confidence intervals of symptoms of depression and anxiety by period (pre-COVID vs. during COVID) and SES-category using OLS-regressions. Our main analysis of inequality across SES is based on education rather than income, for two reasons: First, education has been assessed using the same categories for men and women, allowing for direct comparison between sexes. In contrast, income was assessed using different scales for men and women, and income data is older, dating back to 1999–2009 for women (see “Methods and measures” for more details). Against that background, we use income for additional analyses checking the robustness of our findings. Symptoms of depression and anxiety were measured with the five-item version of the Hopkins Symptom Checklist (SCL-5), a brief and reliable measure of mental distress^22,23^.

Given that respondents could provide multiple responses both in MoBa (i.e., until 2018) and Norflu Coronavirus (during 2020), we calculated the personal average across all available measurements of the Hopkins SCL-5 score within each time period. The OLS-models regress personal average SCL-5 scores on SES-category, a dummy variable indicating time period (before vs. during the COVID-19 pandemic), and the interactions between SES and period. We also control for the age of the respondent in 2020 (in years) using age dummies. By clustering standard errors at the individual level, we adjust inference statistics for multiple observations per respondent. Several additional analyses are provided: (1) Sex-stratified analyses. (2) Sex-stratified analyses using income instead of education as an alternative SES marker to assess the robustness of our findings. (3) A repetition of our main analyses within a subsample of persons who did not report COVID-related employment changes (as opposed to home office, furlough, or job loss) in order to examine whether the pattern observed in the full sample is driven by employment changes induced by the COVID-19 pandemic. (4) An analysis of single items of the SCL-5 to see whether single subdomains like *feeling hopeless about the future* drive the results. (5) An analysis stratifying mental health using a median split based on sex-specific pre-pandemic medians in order to examine to what extent mental health changes associated with the COVID-19 pandemic differ by pre-pandemic mental health. All model results are presented using graphs showing the predicted SCL-5 scores by time-period and SES-category. In addition, we provide tables showing the regression coefficients for the most important models (see Tables A.3 and A.4 in the Supplementary materials).

Furthermore, to examine the sensitivity to model specifications, several robustness analyses were carried out: Firstly, we examined whether a long-term change in mental health inequalities was evolving already before the introduction of measures to mitigate the spread of Sars-COV2 in March 2020. We did this by showing the development of educational inequalities over time, as indicated by intervals of maximum five years before 2020 and single survey waves in 2020. These estimates were obtained using both OLS-regressions and fixed-effects models. The latter examine average individual-level changes in mental health by educational group. Secondly, we examined whether gradients in mental health are comparable across different waves of data collection, both before and during the COVID-19 pandemic. For the pre-COVID period when the time of data collection was largely linked to child age, this analysis holds constant the age of the child that triggered invitation into the study at the time when the survey was conducted. All analysis were conducted using Stata version 16.

## Results

### Increase in depressive symptoms across socioeconomic status

**Figure 2:**
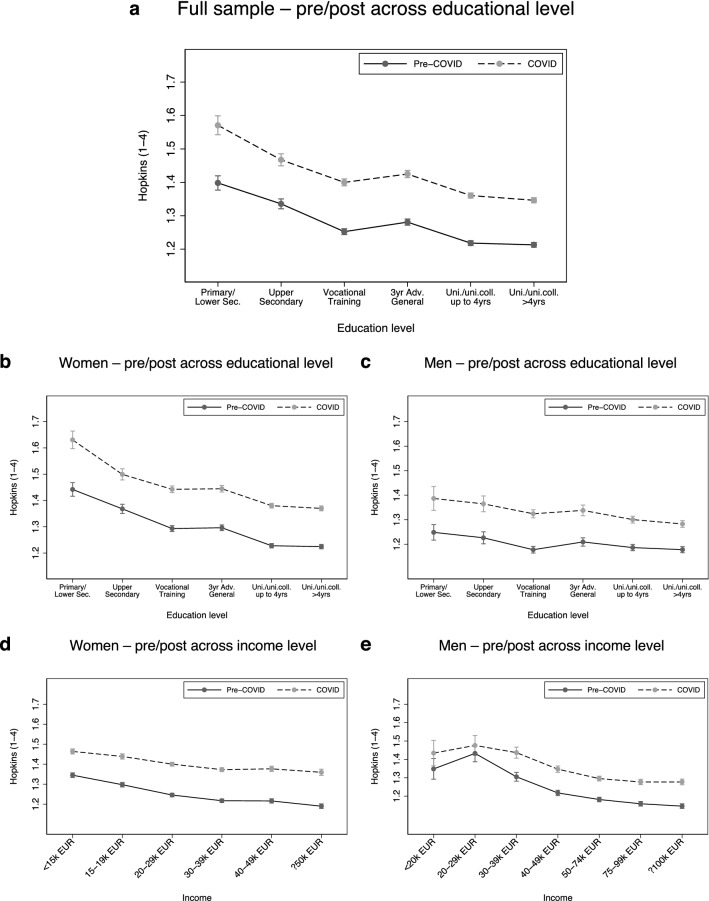
**Average** **levels of symptoms of depression and anxiety by SES and time-period**. The results were obtained from regression models where the individual period-specific average SCL-5 score (pre/during COVID) was regressed on a binary indicator for period (pre-COVID vs. during COVID), an indicator variable for SES (education or income, respectively), and the interaction terms of period and SES. The models also controlled for the age of the respondent in 2020, which is fixed at 46 years in the figures. While the model displayed in Fig. 2a was estimated jointly for women and men, models in Figs. 2b to 2e were estimated separately for women and men. Sample sizes: (**a**) N_persons_ = 111,294, N_obs_ = 202,024; (**b**) N_persons_ = 83,150, N_obs_ = 149,150; (**c**) N_persons_ = 28,144, N_obs_ = 52,874; (**d**) N_persons_ = 80,829, N_obs_ = 145,226; (**e**) N_persons_ = 28,590, N_obs_ = 53,694.

Figure 2, panel a displays educational differences in depressive symptoms before and after the introduction of measures to mitigate the spread of Sars-COV2 in Norway in March 2020 (see Tables S3 and A.4 in the Supplementary materials for the underlying regression models). Two patterns are noteworthy: First, there is a clear SES-gradient in the levels of depressive symptoms prior to the COVID-19 pandemic, i.e., in the 1999–2018 period (the overall negative slope of the solid line). Second, after the introduction of measures to mitigate the spread of Sars-COV2 in 2020, there is a parallel shift upwards (the dashed line)—an increase of comparable size across all educational levels. Hence, there is little evidence that effects of the COVID-19 pandemic on depressive symptoms were unequally distributed across SES. Furthermore, sex-differences in this pattern emerge (Fig. 2b,c): Both the SES-gradient, and the increase in depressive symptoms during the COVID-19 pandemic, are considerably stronger among women. The stronger gradient among women is driven by higher levels of depressive symptoms among women with lower education, while the difference between educational groups is smaller among men. Across all educational levels, the increase in Hopkins SCL-5 scores was about 0.15 scale points among women and about 0.12 scale points among men, corresponding to an approximately 20% smaller increase in depressive symptoms among men (*p* < 0.001). Taken together, this shows that the mental health burden from the COVID-19 pandemic is equally distributed across SES, but somewhat larger for women. As a robustness check, we used income categories instead of education as an indicator of SES. These analyses largely confirm our main result: a similarly sized increase in depressive symptoms across SES as measured by income (Fig. 2d,e). Note that these measures were assessed in different survey waves for men and women (see “Data and Methods” section for details), and that the analysis is underpowered in the lowest income categories among men (see Table A.1 in the Supplementary materials for details).

In the Supplementary materials, we report from a number of robustness checks: First, using OLS- and individual fixed effects regressions, we examined developments in depressive symptoms over time for those with low and high education (Figs. A.1 and A.2). These models show that, although there was an average increase in depressive symptoms in the period 1999–2018, the increase during the first phase of the pandemic was a marked deviation from the time trend. Second, we run our analysis separately for each wave of data collection before and during the COVID-19 pandemic. These results show that absolute differences in mental health between educational groups largely remained similar across different survey waves (Fig. A.3). Third, we examined the role of employment changes during the COVID-19 pandemic by comparing the results from the full sample with a subsample who experienced no change in their employment during COVID-19. Although the increase in depressive symptoms was somewhat smaller among respondents who reported no change in their employment situation, the results in this subsample are consistent with our main result—an increase of comparable size across educational groups (Fig. A.4). Lastly, we ran our analysis separately on each SCL-5 item. This was done to examine whether our results could be driven by items that tap into worries about the future—a worry that might be natural during the early phase of the pandemic. We found an upward shift of similar size for all items, among both men and women (Fig. A.5).

### Increase in depressive symptoms across pre-COVID mental health status

**Figure 3:**
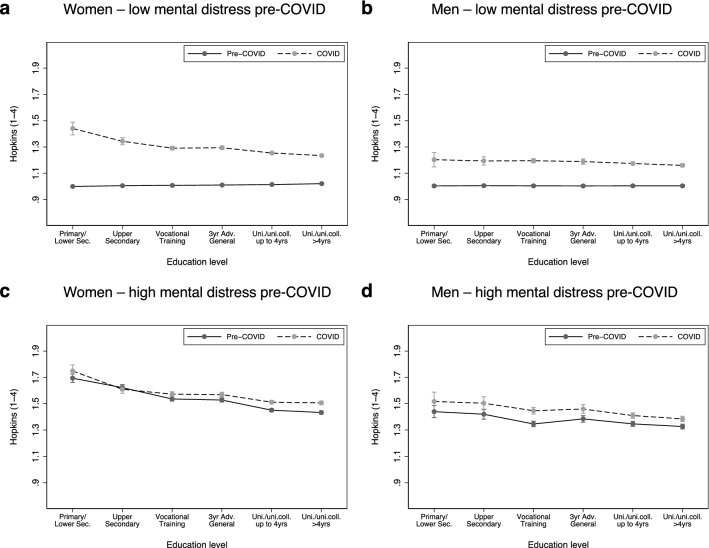
**Average levels of** **symptoms** **of depression** **and anxiety, by mental health status before the COVID-19 pandemic, education, and time-period**. The figures display the estimated mean SCL-5 score, by educational level and mental distress before the COVID-19 pandemic. The results were obtained from regression models where the individual period-specific average SCL-5 score (pre/during COVID) was regressed on a binary indicator for period (pre/during COVID), an indicator variable for education, and the interaction terms of period and education. Separate models were estimated for men and women with high and low levels of mental distress before the COVID-19 pandemic, respectively. Low and high mental distress represent individuals with scores below vs. equal or above the median of the gender-specific pre-COVID Hopkins SCL-5 score. The models also controlled for the age of the respondent in 2020, which is fixed at 46 years in the figures. Sample sizes: (**a**) N_persons_ = 38,730, N_obs_ = 70,469; (**b**) N_persons_ = 13,521, N_obs_ = 25,451; (**c**) N_persons_ = 42,644, N_obs_ = 76,905; (**d**) N_persons_ = 14,507, N_obs_ = 27,307.

Figure 3 displays increases in symptoms of depression and anxiety across education level, analogous to Fig. 2, but stratified on pre-COVID mental health status (median split). Respondents with below-median levels of depressive symptoms prior to the COVID-19 pandemic are shown in panels a and b. Respondents with above-median symptom levels prior to COVID-19 are shown in panels c and d. While the average increases in depressive symptoms in the below-median group were 0.25 for women and 0.17 for men, they were 0.05 and 0.07 in the above-median groups. Also when using the clinical cutoff for stratifying pre-COVID SCL-5 and a balanced sample of respondents participating in *all* relevant study waves, there is evidence of a stronger increase among respondents with fewer depressive symptoms before the pandemic (see Supplementary materials Fig. A.6).

## Discussion and conclusion

Existing literature showed that those with low SES disproportionally carried the burden of economic hardship and health risk during COVID-19. Provided the well-documented impacts of such stressors on mental health, it is surprising that the increases in depressive symptoms observed after the introduction of measures to mitigate the spread of Sars-COV2 were equally strong across socioeconomic strata. Our study cannot explain this counter-intuitive development, but suggests that the mechanisms differ between high and low SES groups. Given that negative employment effects like furlough and associated financial concerns were more frequent among those with *low* SES^24^, our results imply that *high* SES individuals to some extent may have had a stronger response to the increased financial strains, the decline in social contact, the generally increased health risks introduced by COVID-19, or other related factors.

Our results are in line with previous studies suggesting that the mental health burden from the COVID-19 pandemic was equally distributed across SES, but weighing heavier on women^2,14^. While studies from the United Kingdom report that women experienced larger mental health declines during the COVID-19 pandemic than men^25–27^, we are not aware of previous studies that examined changes in mental health by socioeconomic strata separately for men and women. Moreover, our finding is consistent with several studies reporting substantial gender differences in stress response^28–30^: For example, studies find that females are more prone to depression and anxiety, while men struggle more with substance abuse and aggressive behavior^31^. The underlying mechanisms behind such gender differences in stress response are not yet fully understood. Both biological and socialization mechanisms could be involved, and possibly interact. Several studies investigate the role of biological differences in hormonal regulation, in particular the hypothalamic–pituitary–adrenal axis (HPA axis), but the evidence is inconclusive^29^. Other studies suggest that social mechanisms, such as gender specific norms and expectations leading to higher emotional sensitivity and fear avoidance among women, can be important^32^.

We find a greater increase in depressive symptoms among respondents who prior to COVID-19 had better mental health. Our result is in line with a smaller longitudinal study who compared the impact on mental health among respondents with and without pre-existing mental disorders^17^. Our study cannot provide answers regarding the mechanisms behind these results. However, the weaker impact on those with poor mental health could imply that their mental health problems are largely unrelated to external events such as the COVID-19 pandemic. In addition, since mental health is varying over time within individuals, a regression to the mean might account for weak, or even negative, effects among those with the highest symptom levels prior to the COVID-19 pandemic.

An important limitation of this study is that the sample is not representative of the general population in Norway. Survey participants were initially recruited based on pregnancies. Accordingly, the study allows conclusions about the mental health inequalities among parents, but not about the situation of non-parents or persons who became a parent before recruitment started in 1999 or after recruitment ended in 2008, nor about inequalities between parents and non-parents. Therefore, more studies are needed to assess if mental health inequalities developed differently for parents and non-parents during the COVID-19 pandemic, and how the development was in the general population.

## Methods and measures

Symptoms of depression and anxiety were measured with the five-item version of the Hopkins Symptom Checklist (SCL-5), a brief and reliable measure of mental distress^22,23^. Participants used a scale of 1 (not bothered) to 4 (very bothered) to indicate whether they experienced each symptom. The five symptoms are: (1) Feeling fearful, (2) nervousness or shakiness inside, (3) feeling hopeless about the future, (4) feeling blue, (5) worrying too much about things. Responses were averaged across symptoms; scores above 2 are clinically significant. The SCL-5 was administered in each wave of MoBa and rounds 1, 2, 3, and 11 of NorFlu Coronavirus (March 31–April 14, April 14–April 29, April 20–May 12, and August 19–September 1, respectively).

In order to ensure that our conclusions do not depend on specific measures of SES, we used both education and income as indicators. Mothers’ highest level of educational attainment was assessed in the 15th week of pregnancy and when the child was 8 years old, while fathers reported on their education in the 2nd fathers’ survey (2015–2018; see Fig. 1). If a person reported their education at more than one occasion, we used the highest education level reported. Educational categories respond to the following levels: Primary and lower secondary school, upper secondary (1–2 years), vocational training, 3-year advanced general studies (upper secondary), university college or university up to four years, university college or university more than four years. Gross annual income including child support, unemployment benefits and other allowances was reported by mothers in the 15th week of pregnancy (1999–2009) using the following categories: No income, less than 15,000 Euro, 15,000–19,999 Euro, 20,000–29,999 Euro, 30,000–39,999 Euro, 40,000–49,999 Euro, more than 50,000 Euro (original response options used in the survey: No income, less than 150.000 NOK, 151.000–199.999 NOK, 200.000–299.999 NOK, 300.000–399.999 NOK, 400.000–499.999 NOK, more than 500.000 NOK). Fathers reported their gross annual income before taxes in the previous year by assigning themselves to one out of seven categories: Less than 20,000 Euro, 20,000–29,999 Euro, 30,000–39,999 Euro, 40,000–49,999 Euro, 50,000–74,999 Euro, 75,000–99,999 Euro, 100,000 Euro and above (original response options used in the survey: Less than 200,000 NOK, 200,000–299,999 NOK, 300,000–399,999 NOK, 400,000–499,999 NOK, 500,000–749,999 NOK, 750,000–999,999 NOK, 1,000,000 NOK and above). Income of fathers was assessed in the 2nd fathers’ survey (2015–2018).

Age was obtained by subtracting the year of birth from the year when the interview was conducted. Sex was inferred from the respondent identification code, which contained a letter distinguishing between mothers and fathers.

It should be noted that the timing of study participation in the MoBa survey before 2020 depends on the age of the child, not calendar time. As a deviation from that principle, the fathers participated in a second survey in 2015–2018. Importantly, this leads to significant differences in gender composition over time in the data. Besides our substantial interest in gender differences, this pattern makes estimation of joint time trends for men and women challenging and warrants gender-specific analyses.

Information about changes in employment status due to the COVID-19 pandemic was used in additional analyses. No change in employment is defined as not reporting a change in the employment situation in the first and second round of NorFlu Coronavirus (as opposed to reporting home office, temporary layoff, or job loss; note that home office was only introduced as a response option from wave 2 and onwards). If a respondent reported any employment change due to the COVID-19 pandemic or if this information was missing in round 3 or 11, observations for this and possible subsequent time points were set to missing.

### Ethics approval

MoBa and the NorFlu Coronovirus Study are conducted according to the guidelines laid down in the Declaration of Helsinki, and written informed consent was obtained from all participants when entering the MoBa study. Ethics approval for the project “Using MoBa to understand the Covid-19 pandemic” was obtained from the Regional Committee for Medical Research Ethics South-Eastern Norway (REK; approval no. 127708 for MoBa, and no. 18403 for NorFlu).

## Supplementary Information

Supplementary Information.

## Data Availability

The consent given by the participants does not open for storage of data on an individual level in repositories or journals. Researchers who want access to data sets for replication should submit an application to datatilgang@fhi.no. Access to data sets requires approval from The Regional Committee for Medical and Health Research Ethics in Norway and an agreement with MoBa.
