# Supplementary material for: Longitudinal evidence on the development of socioeconomic inequalities in mental health due to the COVID-19 pandemic in Norway
Source: Sci Rep. 2022 Mar 9;12:3837. doi: 10.1038/s41598-022-06616-7 (PMC8907231; doi:10.1038/s41598-022-06616-7)
Supplement: Supplementary file 1 — Supplementary Information. [file 41598_2022_6616_MOESM1_ESM.pdf]

Reme et al.: Longitudinal evidence on the development of socioeconomic inequalities in mental health due to the COVID-19 pandemic in Norway (Scientific Reports)

Supplementary materials

Contents:

- Table A.1: Descriptive statistics for the analytical sample
- Table A.2: Overview of raw data (MoBa- and NorFlu-surveys)
- Table A.3: Regression of symptoms of depression and anxiety on period and education – age controlled (Figures 1a, 1b and 1c)
- Table A.4: Regression of symptoms of depression and anxiety on period and income – age controlled (Figures 1d and 1e)
- Figure A.1: Development in depressive symptoms across gender
- Figure A.2: Development in depressive symptoms across gender (fixed effects models)
- Figure A.3: Gradients by survey wave
- Figure A.4: Full sample vs. persons without COVID-induced employment change
- Figure A.5: Models for single Hopkins SCL-5 items
- Figure A.6: Average levels of depressive symptoms, by mental health status before the COVID-19 pandemic

Table A.1: Descriptive statistics for the analytical sample

	**Full Sample** Mean (SD) / %	**Women** Mean (SD) / %	**Men** Mean (SD) / %
Hopkins SCL-5 score	1.30 (0.39)	1.33 (0.41)	1.23 (0.34)
Age in 2020 (years)	46.43 (5.27)	45.77 (4.99)	48.30 (5.56)
Male	26	0	100
High education (dummy)	68	68	69
Educational level			
Primary and lower secondary	2	2	2
Upper secondary	4	4	4
Vocational training	14	12	18
3-years advanced general studies	12	13	8
University or university college up to 4 years	36	37	35
University or university more than 4 years	32	31	34
Income women			
<15k Euro		15	
15-19k Euro		11	
20-29k Euro		34	
30-39k Euro		27	
40-49k Euro		8	
≥50k Euro		5	
Income men			
<20k Euro			1
20-29k Euro			1
30-39k Euro			5
40-49k Euro			14
50-74k Euro			41
75-99k Euro			21
≥100k Euro			17
Persons	111,294	83,150	28,144
Observations	202,024	149,150	52,874

Note. High education = University or university college up to four years *or* University or university college more than four years (vs. all lower levels of education). Due to missing values, information on income is based on slightly different samples: Women: $N_{persons}$=80,829, N_{obs}=145,226, Men: $N_{persons}$=28,590, N_{obs}=53,694.

Table A.2: Overview of raw data (MoBa- and NorFlu-surveys)

Survey wave	Respondent	Time period	$N_{respondents}$
Pregnancy, week 15	Mother	1999-2009	86,391
5-year interview	Mother	2005-2014	36,824
8-year interview	Mother	2003-2017	37,899
1st interview	Father	2000-2009	66,975
2nd interview	Father	2015-2018	29,307
COVID round 1	Both	March 2020	114,489
COVID round 2	Both	April 2020	109,870
COVID round 3	Both	May 2020	101,744
COVID round 11	Both	Aug 2020	61,026

Note. The table provides an overview of the survey waves from which the analytical sample was selected. The pre-COVID waves are above the line. The table indicates the time period of data collection, whether the survey was aimed at mothers or fathers, and the number of participating individuals in a given wave.

Table A.3: Regression of symptoms of depression and anxiety on period and *education* – age controlled (Figures 1a, 1b and 1c)

	All	Men	Women
COVID (vs. before COVID)	0.173***	0.138***	0.189***
	(0.0149)	(0.0224)	(0.0181)
Education (reference: Primary and lower secondary)			
Upper secondary	-0.0626***	-0.0222	-0.0738***
	(0.0124)	(0.0184)	(0.0149)
Vocational	-0.146***	-0.0708***	-0.149***
	(0.0108)	(0.0154)	(0.0131)
Upper secondary	-0.117***	-0.0391*	-0.146***
	(0.0109)	(0.0162)	(0.0131)
Uni low	-0.180***	-0.0621***	-0.214***
	(0.0105)	(0.0152)	(0.0126)
Uni high	-0.185***	-0.0706***	-0.218***
	(0.0105)	(0.0152)	(0.0126)
*COVID (vs. before COVID) * education*			
COVID * Upper secondary	-0.0407*	0.0000431	-0.0574**
	(0.0176)	(0.0270)	(0.0214)
COVID * Vocational	-0.0250	0.00831	-0.0392*
	(0.0155)	(0.0232)	(0.0190)
COVID * Upper secondary	-0.0286	-0.00961	-0.0402*
	(0.0156)	(0.0241)	(0.0189)
COVID * Uni low	-0.0302*	-0.0242	-0.0362*
	(0.0151)	(0.0228)	(0.0183)
COVID * Uni high	-0.0391**	-0.0336	-0.0431*
	(0.0151)	(0.0227)	(0.0183)
Constant	1.644***	1.221***	1.667***
	(0.0654)	(0.105)	(0.0674)
Persons	111,294	28,144	83,150
Observations	202,024	52,874	149,150

Standard errors in parentheses
* $p < 0.05$, ** $p < 0.01$, *** $p < 0.001$

Note. The table shows the results from OLS-regressions corresponding to Figures 1a, 1b and 1c. The models use clustered standard errors on the level of individuals, with the Hopkins SCL-5 score as the dependent variable. The models also control for respondent age in 2020 using dummy variables for each age.

Table A.4: Regression of symptoms of depression and anxiety on period and *income* – age controlled (Figures 1d and 1e)

	Men	Women
COVID (vs. before COVID)	0.0861^{*}	0.119^{***}
	(0.0354)	(0.00542)
Income (reference groups:		
men: <20k Euro */ women:* <15k Euro*)*		
20-29k Euro / 15-19k Euro	0.0845^{*}	-0.0476^{***}
	(0.0360)	(0.00598)
30-39k Euro / 20-29k Euro	-0.0430	-0.0993^{***}
	(0.0299)	(0.00477)
40-49k Euro / 30-39k Euro	-0.131^{***}	-0.128^{***}
	(0.0284)	(0.00479)
50-74k Euro / 40-49k Euro	-0.167^{***}	-0.129^{***}
	(0.0281)	(0.00582)
75-99k Euro / ≥50k Euro	-0.190^{***}	-0.155^{***}
	(0.0281)	(0.00621)
≥100k Euro	-0.203^{***}	-
	(0.0282)	
*COVID (vs. before COVID) * income*		
COVID * 20-29k Euro / 15-19k Euro	-0.0427	0.0226^{**}
	(0.0456)	(0.00788)
COVID * 30-39k Euro / 20-29k Euro	0.0457	0.0357^{***}
	(0.0382)	(0.00616)
COVID * 40-49k Euro / 30-39k Euro	0.0432	0.0367^{***}
	(0.0361)	(0.00620)
COVID * 50-74k Euro / 40-49k Euro	0.0280	0.0419^{***}
	(0.0356)	(0.00776)
COVID * 75-99k Euro / ≥50k Euro	0.0326	0.0507^{***}
	(0.0357)	(0.00866)
COVID * ≥100k Euro	0.0457	-
	(0.0358)	
Constant	1.295^{***}	1.614^{***}
	(0.141)	(0.0920)
Persons	28,590	80,829
Observations	53,694	145,226

Standard errors in parentheses
* $p < 0.05$, ** $p < 0.01$, *** $p < 0.001$

Note. The table shows the results from OLS-regressions corresponding to Figures 1d and 1e. The models use clustered standard errors on the level of individuals, with the Hopkins SCL-5 score as the dependent variable. The income categories for men and women assessed in different survey waves and differ between sexes. This is indicated by the forward slash (/) in the table - the categories for men are written first, while the categories for women follow after the forward slash. The models also control for respondent age in 2020 using dummy variables for each age. k = 1000.

Figure A.1: Development in depressive symptoms across gender

(a) Women

(b) Men

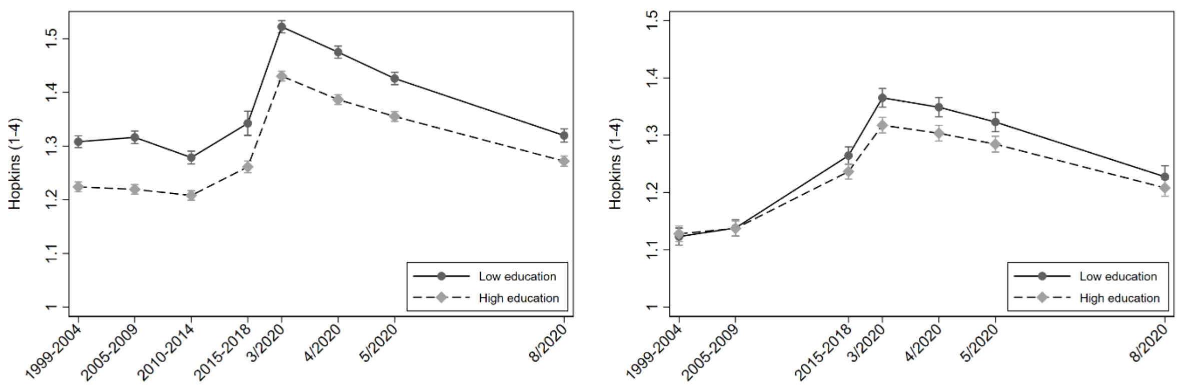

Note. The figures display the estimated mean SCL-5 score in different time periods, by education. Education level was median split, with high education representing respondents having a university degree. The results were obtained from OLS-regression models where the individual time-specific average SCL-5 score (by calendar-time interval) was regressed on an indicator variable for period, an indicator variable for education, and interaction terms for period and education. The models also control for the age of the respondent in 2020, which is fixed at 46 years in the figures (Figure A.1a: $N_{persons}$=83,150, N_{obs}=364,767; Figure A.1b: $N_{persons}$=28,143, N_{obs}=132,851). Figure A.1b excludes the 2010-2014 period because it only includes 18 observations from men.

Figure A.2: Development in depressive symptoms across gender (fixed effects models)

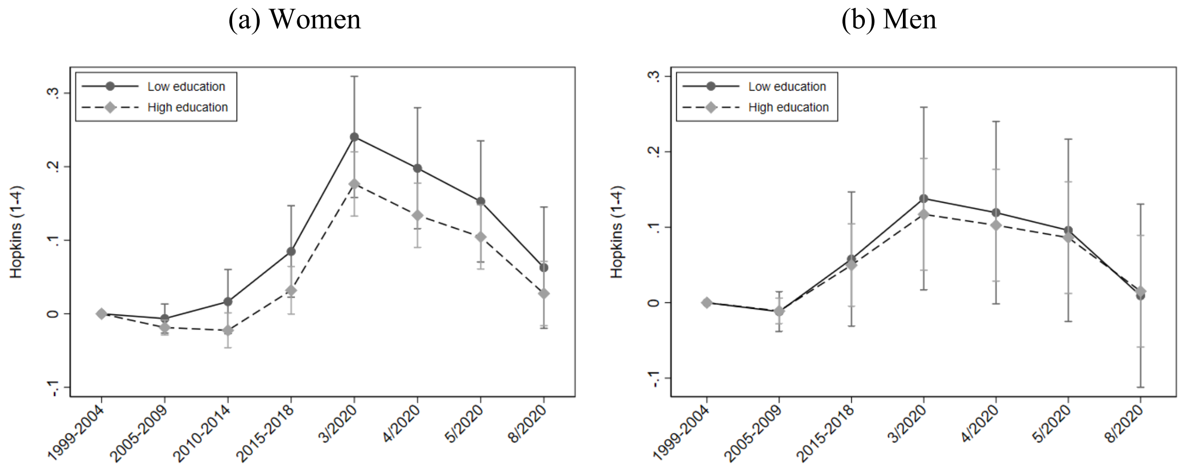

Note. The figures show results from individual fixed effects models, hence relying only on within-person changes in SCL-5 over time relative to the 1999-2004 period. Accordingly, only respondents with valid responses at two or more time-periods displayed in the figures are included in the analysis. Estimates were obtained from separate models for low and high education, with the latter indicating having obtained a university degree. Models include an indicator for time-period and control for age using dummy variables. In case respondents participated in multiple surveys during a period, the average SCL-5 and average age within that period were used. For men, the 2010-2014 period was omitted, because it only included 17 observations (Figure A.2a: $N_{persons}$=73,431, N_{obs}=355,048; Figure A.2b: $N_{persons}$=27,692, N_{obs}=132,400).

Figure A.3: Gradients by survey wave

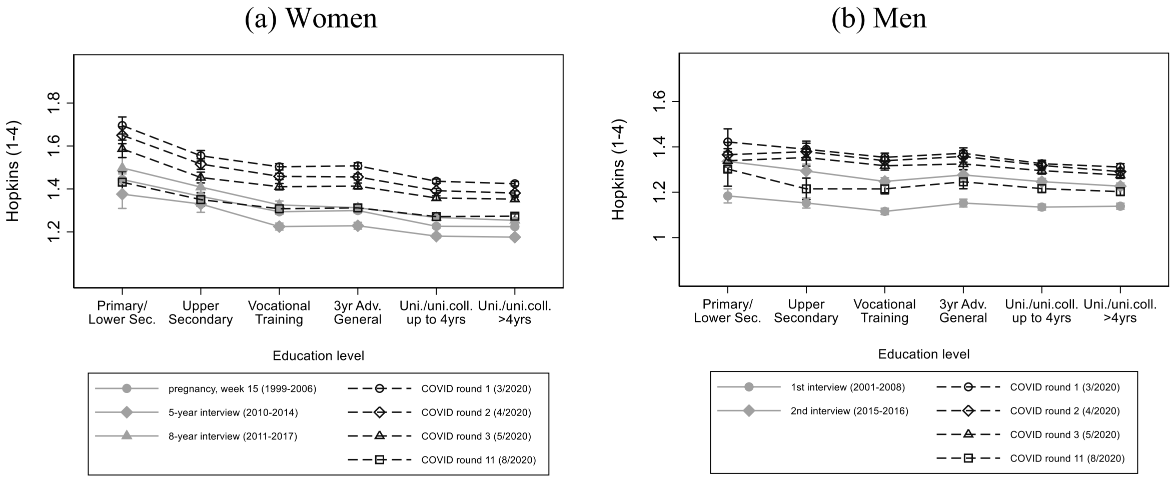

Note. The figures display the estimated mean SCL-5 score by education, stratifying assessments into different survey waves. Except for the 2nd interview with fathers and the interviews during COVID, the timing of the survey was largely based on the child(ren) that triggered study enrolment of the parents. The results were obtained from regression models where the individual, survey-specific average SCL-5 score was regressed on the survey indicator interacted with educational attainment. The models also controlled for age of the respondent, which is fixed at 46 years in the figures (Figure A.3a: $N_{persons}$=83,150, N_{obs}=372,542; Figure A.3b: $N_{persons}$=28,144, N_{obs}=132,347).

Figure A.4: Full sample vs. persons without COVID-induced employment change

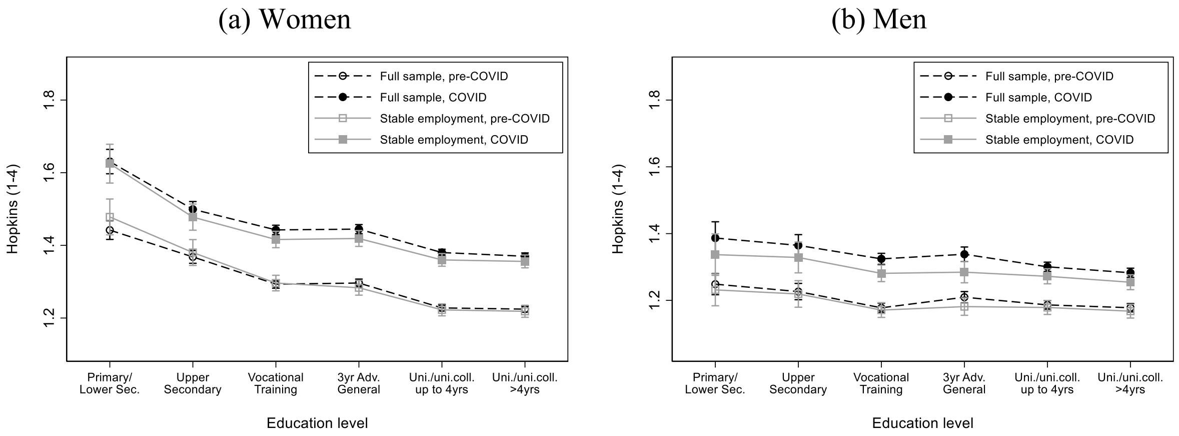

Note. The figures display the estimated mean SCL-5 score by education and time, for the full sample and for persons who reported that their employment did not change due to the COVID-19 pandemic. The results were obtained from regression models where the individual period-specific average SCL-5 score (pre/during COVID) was regressed on a binary indicator for period (pre-COVID vs. during COVID), an indicator variable for education level, and the respective interaction terms. The models also control for age of the respondent, which is fixed at 46 years in the figures. No change in employment is defined as not reporting a change in the employment situation in the first and second round of NorFlu Coronavirus (as opposed to reporting home office, temporary layoff, or job loss; note that home office was only introduced as a response option from wave 2 and onwards). If a respondent reported any employment change due to the COVID-19 pandemic or if this information was missing in round 3 or 11, observations for this and possible subsequent time points were set to missing. Sample sizes: Women, full sample: $N_{persons}$=83,150, N_{obs}=149,150; Women, "no change" sample: $N_{persons}$=21,998, N_{obs}=43,473; Men, full sample: $N_{persons}$=28,144, N_{obs}=52,874; Men, "no change" sample: $N_{persons}$=9,202, N_{obs}=18,369.

Figure A.5: Models for single Hopkins SCL-5 items

Item 1: Feeling fearful

(a) Women

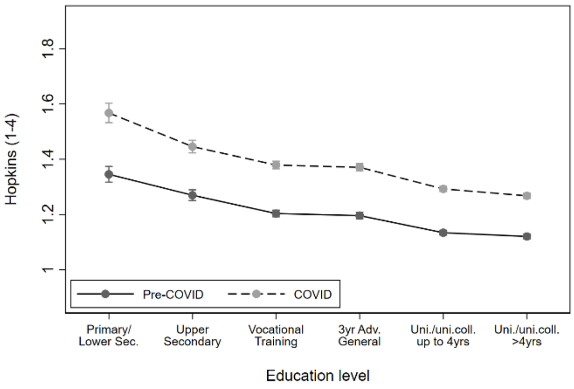

(b) Men

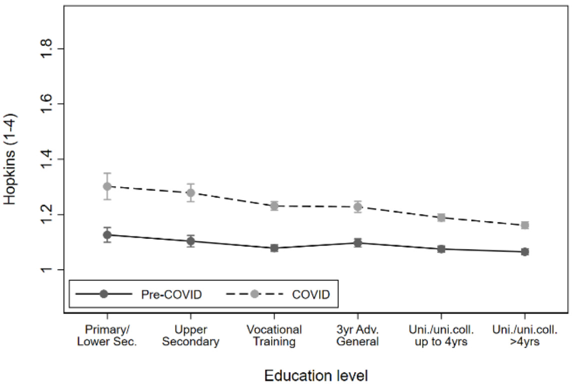

Item 2: Nervousness or shakiness inside

(c) Women

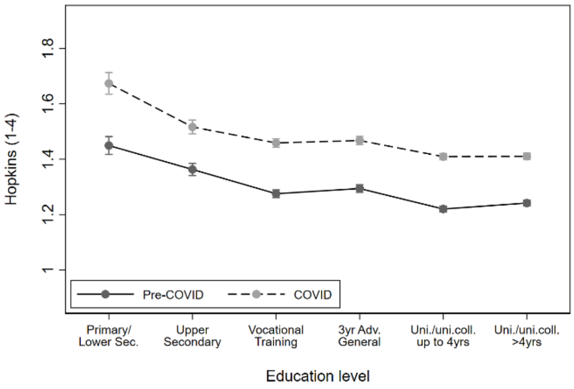

(d) Men

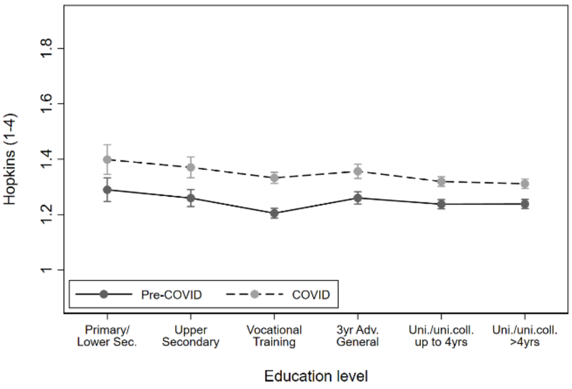

Item 3: Feeling hopeless about the future

(e) Women

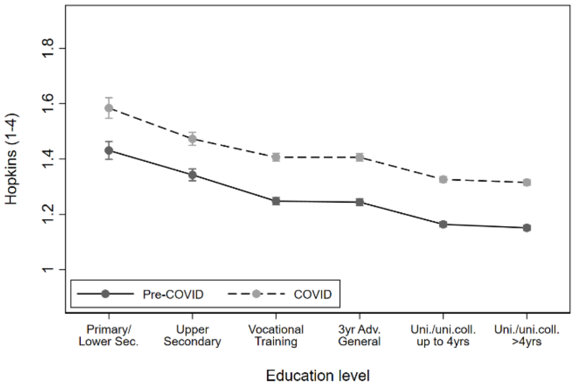

(f) Men

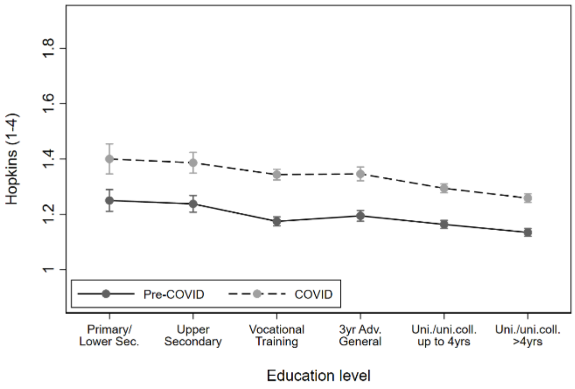

Item 4: Feeling blue

(g) Women

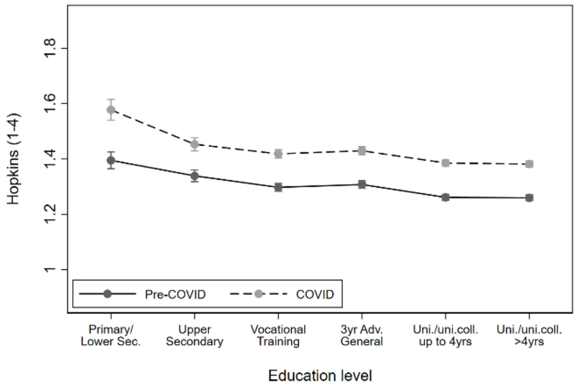

(h) Men

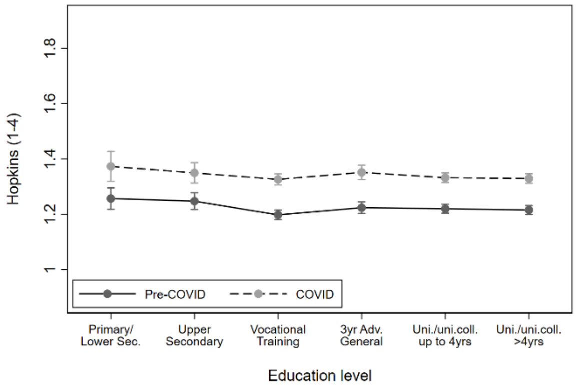

Item 5: Worrying too much about things

(i) Women

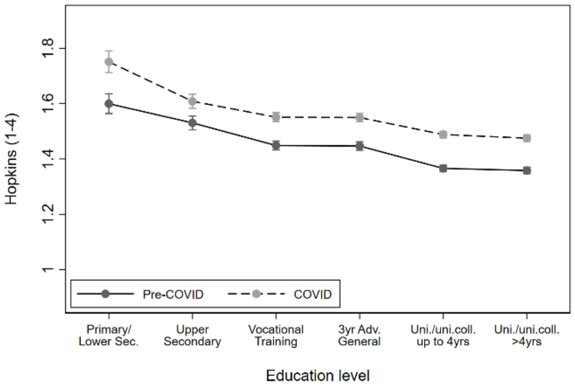

(j) Men

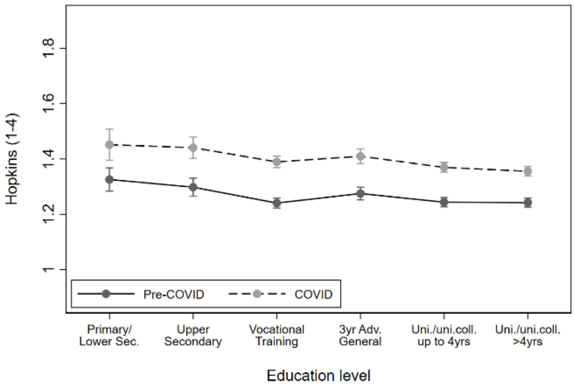

Note. The figures display the estimated mean SCL-5 score by socioeconomic status, separately for each of the five items of the SCL-5. The results were obtained from regression models where the individual period-specific average SCL-5 score (pre/during COVID) was regressed on a binary indicator for period (pre-COVID vs. during COVID),

an indicator variable for education level, and the respective interaction terms. The models also control for age of the respondent, which is fixed at 46 years in the figures. Sample sizes are equal across items, as the analysis were limited to observations where all five items of the SCL-5 were non-missing (Women: $N_{persons}$=83,150, N_{obs}=149,150; Men: $N_{persons}$=28,144, N_{obs}=52,874).

Figure A.6: Average levels of depressive symptoms, by mental health status before the COVID-19 pandemic

(a) Women – low mental distress pre-COVID

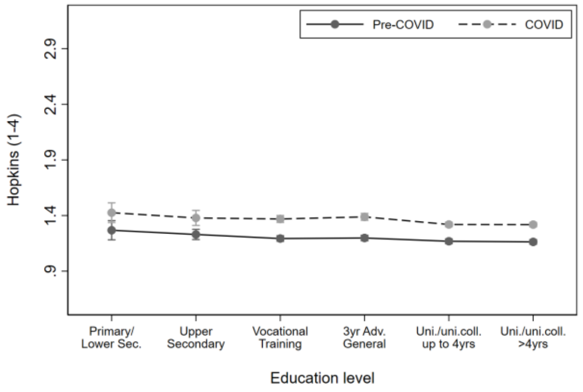

(b) Men – low mental distress pre-COVID

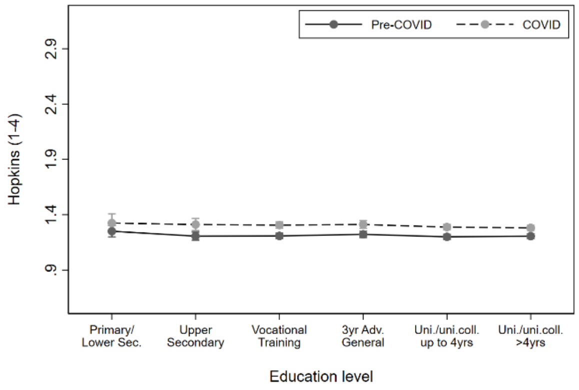

(c) Women – high mental distress pre-COVID

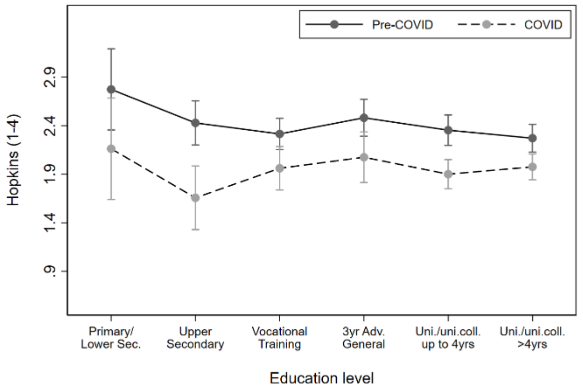

(d) Men – high mental distress pre-COVID

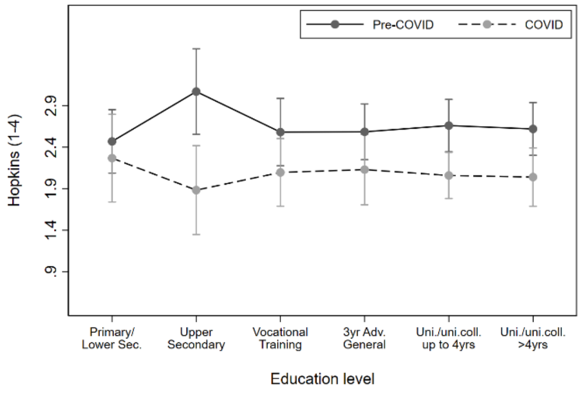

Note. The figures display the estimated mean SCL-5 score, by educational and period for men and women with pre-COVID depressive symptom levels below/equal vs. above the cutoff for clinical relevance of the Hopkins SCL-5 (cutoff = 2). The sample for this analysis was restricted to participants providing valid measures in all relevant study waves (before the COVID-19 pandemic: two and three observations for men and women, respectively; during the COVID-19 pandemic: four observations). The results were obtained from regression models where the individual survey specific average SCL-5 score was regressed on a binary indicator for period (pre-COVID vs. during COVID), an indicator variable for education, and the interaction terms of period and education. Separate models were estimated for men and women below and above the cutoff, respectively. The models also controlled for the age of the respondent in 2020, which is fixed at 46 years in the figures. Sample sizes: Figure 3a: $N_{persons}$=13,671, N_{obs}=95,697; Figure 3b: $N_{persons}$=8,941, N_{obs}=53,646; Figure 3c: $N_{persons}$=345, N_{obs}=2,415; Figure 3d: $N_{persons}$=154, N_{obs}=924.
